# Evaluation of improved attenuation correction in whole-body PET/MR on patients with bone metastasis using various radiotracers

**DOI:** 10.1007/s00259-020-04738-6

**Published:** 2020-03-03

**Authors:** Hong Grafe, Maike E. Lindemann, Verena Ruhlmann, Mark Oehmigen, Nader Hirmas, Lale Umutlu, Ken Herrmann, Harald H. Quick

**Affiliations:** 1 Department of Nuclear Medicine, University Hospital Essen, University Duisburg-Essen, Hufelandstr. 55, 45147 Essen, Germany; 2 High-Field and Hybrid MR Imaging, University Hospital Essen, University Duisburg-Essen, Hufelandstr. 55, 45147 Essen, Germany; 3 Department of Diagnostic and Interventional Radiology and Neuroradiology, University Hospital Essen, University Duisburg-Essen, Hufelandstr. 55, 45147 Essen, Germany; 4 Erwin L. Hahn Institute for Magnetic Resonance Imaging, University Duisburg-Essen, Zollverein, 45141 Essen, Germany

**Keywords:** Whole-body PET/MR, Bone metastasis, Attenuation correction, Truncation correction, HUGE, MLAA

## Abstract

**Purpose:**

This study evaluates the quantitative effect of improved MR-based attenuation correction (AC), including bone segmentation and the HUGE method for truncation correction in PET/MR whole-body hybrid imaging specifically of oncologic patients with bone metastasis and using various radiotracers.

**Methods:**

Twenty-three patients that underwent altogether 28 whole-body PET/MR examinations with findings of bone metastasis were included in this study. Different radiotracers (^18^F-FDG, ^68^Ga-PSMA, ^68^Ga-DOTATOC, ^124^I–MIBG) were injected according to appropriate clinical indications. Each of the 28 whole-body PET datasets was reconstructed three times using AC with (1) standard four-compartment μ-maps (background air, lung, muscle, and soft tissue), (2) five-compartment μ-maps (adding bone), and (3) six-compartment μ-maps (adding bone and HUGE truncation correction). The SUV_max_ of each detected bone lesion was measured in each reconstruction to evaluate the quantitative impact of improved MR-based AC. Relative difference images between four- and six-compartment μ-maps were calculated. MR-based HUGE truncation correction was compared with the PET-based MLAA truncation correction method in all patients.

**Results:**

Overall, 69 bone lesions were detected and evaluated. The mean increase in relative difference over all 69 lesions in SUV_max_ was 5.4 ± 6.4% when comparing the improved six-compartment AC with the standard four-compartment AC. Maximal relative difference of 28.4% was measured in one lesion. Truncation correction with HUGE worked robust and resulted in realistic body contouring in all 28 exams and for all 4 different radiotracers. Truncation correction with MLAA revealed overestimations of arm tissue volume in all PET/MR exams with ^18^F-FDG radiotracer and failed in all other exams with radiotracers ^68^Ga-PSMA, ^68^Ga-DOTATOC, and ^124^I- MIBG due to limitations in body contour detection.

**Conclusion:**

Improved MR-based AC, including bone segmentation and HUGE truncation correction in whole-body PET/MR on patients with bone lesions and using various radiotracers, is important to ensure best possible diagnostic image quality and accurate PET quantification. The HUGE method for truncation correction based on MR worked robust and results in realistic body contouring, independent of the radiotracers used.

## Introduction

Whole-body positron emission tomography/magnetic resonance (PET/MR) hybrid imaging has gained momentum in oncologic applications during the last decade, given the unique ability to combine excellent soft tissue contrast and other functional imaging parameters provided by MR imaging with the quantification of radiotracer metabolism provided by PET [1].

While promising, many physical and practical challenges have been subject to interdisciplinary research aiming to further develop hybrid imaging with PET/MR for specific clinical applications. For example, attenuation correction (AC) of human soft tissues in integrated PET/MR has to rely on MR imaging to allow quantification of PET imaging, as attenuation information from computed tomography (CT) is missing [2, 3]. MR images mainly provide proton densities and tissue-dependent spin relaxation properties and cannot be directly converted to linear attenuation coefficients (LAC) at 511 keV as needed for AC of PET data. In addition, the inherently limited field-of-view (FOV) in MRI may lead to truncation artifacts along the patients’ arms in MR-based AC, potentially resulting in a bias in PET quantification [4]. Further, the substitution of bone as soft tissue may lead to a systematical underestimation in PET signal [5]. These and other issues have been subject to thorough research to further develop and improve MR-based AC in PET/MR.

The established standard MR-based AC for the Biograph mMR integrated whole-body PET/MR system (Siemens Healthcare GmbH, Erlangen, Germany) is a Dixon-VIBE MR sequence that provides tissue segmentation for four different tissue compartments (background air, lung, fat, soft tissue) with predefined linear attenuation coefficients [6]. Recently, an improved version of MR-based AC has been released by the manufacturer that now features improved spatial resolution of the Dixon-VIBE MR sequence due to image acquisition acceleration with the CAIPIRINHA method [7]. Additionally, the manufacturer included a bone model approach [8, 9], and additional truncation correction by the MR-based HUGE method [10, 11] to the most current version of the attenuation correction method.

The improved MR-based AC method including bone model and MR-based truncation correction with the HUGE method has recently been evaluated in a clinical study using PET/MR whole-body imaging on patients that have been injected ^18^F-FDG as radiotracer [12]. The main result of this study was that the improved MR-based AC version provides robust and accurate quantification in patients injected with ^18^F-FDG. Similar results were reported by Lindemann et al. [11] who specifically examined two most current implementations for truncation correction, namely the MR-based HUGE method and a PET-based algorithm for truncation correction, e.g., the maximum likelihood estimation of activity and attenuation (MLAA) [13–15] on patients injected with ^18^F-FDG. The results of the study by Lindemann et al. indicated that the MR-based HUGE method works robust and provides accurate body contour detection for truncation correction in each patient case [11]. This was also the case for HUGE in one patient where ^124^I instead of ^18^F-FDG was injected as radiotracer. In this specific case, truncation correction by MLAA failed due to the fact that the PET-based algorithm did not find enough signal from unspecific tracer accumulation in tissue to derive body contours [11].

Against this background, the present study was conducted to evaluate the quantitative effect of the improved MR-based AC method, including bone segmentation and HUGE truncation correction, in PET/MR whole-body hybrid imaging, specifically on oncologic patients with bone metastasis and using various radiotracers.

## Materials and methods

### Patient population

Patients with findings of bone metastasis on PET/MR were included in this retrospective study. Twenty-three patients with altogether 28 whole-body PET/MR examinations according to a whole-body standard clinical protocol were included in the study. The mean age was 56 years (range 17–83 years) and the mean body mass index (BMI) was 22.8 kg/m^2^ (range 15.5–32.3 kg/m^2^) with 17 male and 6 female patients. Depending on the clinical indication and primary tumor finding, different radiotracers were injected. Fluoro-18-fluorodeoxyglucose (^18^F-FDG) was injected in 17 PET/MR examinations, gallium-68-prostate specific membrane antigen (^68^Ga-PSMA) injected in five examinations, gallium-68-dotatate (^68^Ga-Dotatoc) was injected in five PET/MR examinations, and iodine-124-metaiodobenzylguanidine (^124^I-MIBG) was injected in one exam. The average injected activity was 236.6 ± 46.2 MBq for ^18^F-FDG, 109.0 ± 30.1 MBq for ^68^Ga-PSMA, 92.2 ± 10.8 MBq for ^68^Ga-Dotatoc, and 47.7 MBq for ^124^I-MIBG. The average tracer post-injection time to start PET/MR scanning was 81 ± 29 min for ^18^F-FDG, 140.2 ± 54.4 min for ^68^Ga-PSMA, 64.8 ± 23 min for ^68^Ga-Dotatoc, and 24 h for ^124^I-MIBG. Further information about the patient population is listed in Table 1. Written informed consent was given before conducting the PET/MR examination, and the approval of the ethical board was obtained. All measurements were performed in accordance with the ethical standards of the institutional review board of the Medical Faculty of the University Duisburg-Essen and with the principles of the 1964 Declaration of Helsinki and its later amendments.

**Table 1:** Detailed patient and examination information sorted according to injected tracer

Patient no.	Exam no.	Age (years)	Sex	BMI (kg/m^2^)	Tracer	Injected activity (MBq)	Scan start (min. after injection)	Type of malignancy
1	1	62	F	25.6	18F-FDG	261	58	Breast cancer
2	2	35	M	20.0	18F-FDG	228	57	Myxoid liposarcoma
3	3	74	M	23.9	18F-FDG	214	120	Melanoma
	4	74	M	23.9	18F-FDG	214	60	Melanoma
4	5	53	M	21.9	18F-FDG	229	70	Breast cancer
5	6	76	F	22.9	18F-FDG	202	111	Osteosarcoma
6	7	83	F	17.0	18F-FDG	185	154	Thyroid cancer
7	8	65	M	26.0	18F-FDG	334	89	Melanoma
8	9	63	F	2.3	18F-FDG	300	67	Sarcoma
9	10	30	M	17.1	18F-FDG	227	57	Postradiogenic Leiomyosarcoma
10	11	17	F	19.0	18F-FDG	231	56	Adrenocortical carcinoma
11	12	66	M	20.5	18F-FDG	250	88	Hepatocellular carcinoma
	13	66	M	20.8	18F-FDG	267	53	Hepatocellular carcinoma
12	14	17	F	16.1	18F-FDG	178	103	Rhabdomyosarcoma
	15	17	F	15.8	18F-FDG	210	57	Rhabdomyosarcoma
	16	17	F	15.5	18F-FDG	170	67	Rhabdomyosarcoma
13	17	54	M	25.1	18F-FDG	322	118	Lung cancer
**Mean ± SD**		**51 ± 22**	**6 F, 7 M**	**20.8 ± 3.5**		**236.6 ± 46.2**	**81 ± 29**	
14	18	66	M	23.1	[68Ga]-PSMA	145	84	Prostate carcinoma
15	19	75	M	32.3	[68Ga]-PSMA	95	153	Prostate carcinoma
16	20	77	M	25.3	[68Ga]-PSMA	145	67	Prostate carcinoma
17	21	50	M	23.3	[68Ga]-PSMA	85	307	Prostate carcinoma
18	23	54	M	21.7	[68Ga]-PSMA	75	90	Prostate carcinoma
**Mean ± SD**		**64 ± 21**	**5 M**	**25.1 ± 5.3**		**109 ± 30.1**	**140.2 ± 54.4**	
19	22	25	M	25.0	[68Ga]-DOTATOC	110	55	Paraganglioma
20	24	77	M	21.0	[68Ga]-DOTATOC	78	109	Neuroendocrine tumor
21	25	78	M	20.1	[68Ga]-DOTATOC	97	62	Neuroendocrine tumor
22	26	71	M	24.1	[68Ga]-DOTATOC	90	40	Neuroendocrine tumor
23	27	67	M	22.8	[68Ga]-DOTATOC	86	58	Pheochromocytoma
**Mean ± SD**		**63 ± 19**	**5 M**	**22.6 ± 1.8**		**92.2 ± 10.8**	**64.8 ± 23**	
23	28	66	M	24.4	124I-MIBG	47.7	24 h	Pheochromocytoma

### Imaging system

All patient examinations were performed on an integrated PET/MR whole-body hybrid imaging system (Biograph mMR, Siemens Healthcare GmbH, Erlangen, Germany) [1*,*^16^]. The version of the PET/MR operating system at the time of this study was *syngo* MR VE11P (Siemens Healthcare GmbH).

### Attenuation correction

All AC maps (μ-maps) were applied to perform image-based AC and scatter correction. The patient-specific tissue AC and scatter correction in this study is based on an improved high-resolution Dixon-VIBE sequence with CAIPIRINHA parallel imaging acceleration [7], which provides higher spatial resolution and faster data acquisition compared to previous AC sequence implementations [4*,*^6^]. The 3D Dixon-VIBE MR sequence is acquired in axial orientation in five bed positions to cover the patient from the skull to the upper legs.

The Dixon-VIBE sequence protocol used for AC in this implementation utilizes the following scan parameters: parallel imaging acceleration factor *R* = 5, matrix 390 × 240 with 1.3 × 1.3 mm in-plane pixel size, 136 slices each 3.0 mm, flip angle 10°, repetition time (TR)  3.8 ms, echo times (TE) TE1/TE2 1.2/2.4 ms, and acquisition time (TA) for each of the five bed positions was 19 s. MRI-based image information of the patient provided by the Dixon-VIBE sequence is then automatically segmented into four tissue compartments that are each assigned the specific linear LACs for soft tissue (0.1 cm^−1^), fat (0.0854 cm^−1^), lung (0.0224 cm^−1^), and background air (0.0 cm^−1^). This sequence providing four tissue compartments for AC is referred to henceforth as the four-compartment μ-map, and it serves as a reference in this study [1, 6, 17].

The new *syngo* MR E11P software features reconstruction of bone data as a fifth compartment from the high-resolution CAIPIRINHA Dixon-VIBE sequence. The reconstruction applies a model-based bone segmentation algorithm [8, 9, 18] and adds the major bones (skull, spine, pelvic bones, upper femurs) as a fifth compartment to the previous mentioned four-tissue compartment (air, lung, muscle, soft tissue, bone). The model-based method assigns continuous LACs to bone from 0.1 up to 0.2485 cm^−1^. Detailed information on this bone model is provided by Paulus et al. [8].

**Fig. 1:**
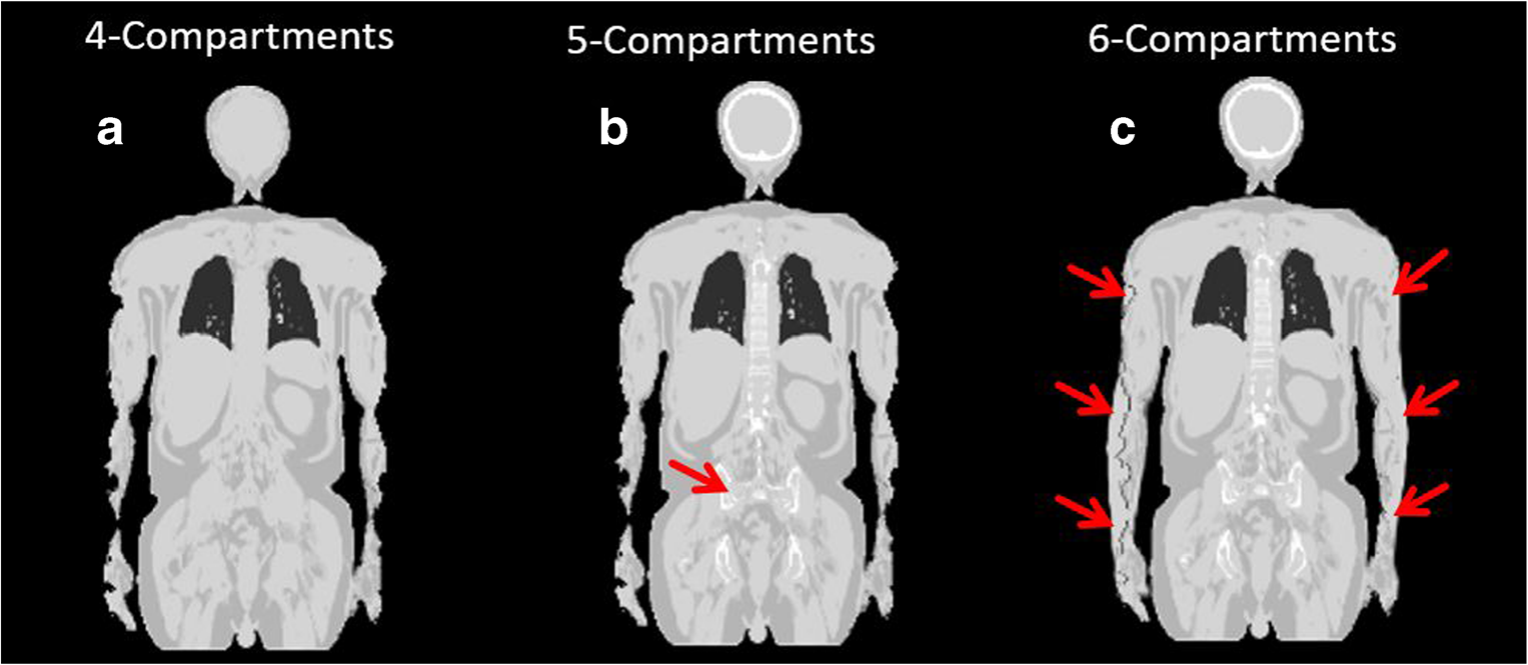
Segmentation from the improved CAIPIRINHA Dixon-VIBE sequence including bone model and MR-based truncation correction (HUGE). Example images of patient μ-maps: **a** four-compartment (background air, lung, muscle, soft tissue), **b** five-compartment with marked (red arrow) bone model (four-compartment plus bone), **c** six-compartment (four-compartment plus bone and HUGE truncation correction). The impact of HUGE truncation correction is marked with red arrows (**c**)

The segmentation from the CAIPIRINHA Dixon-VIBE sequence with added bone model and truncation correction with the HUGE method is referred to henceforth as the six-compartment μ-map (background air, lung, muscle, soft tissue, bone, HUGE) (Fig. 1) [12].

In conventional MR imaging, the MR imaging FOV is often limited to a spherical diameter of about 45–50 cm around the isocenter of the MRI system. Tissues exceeding this volume may be displayed with geometric distortions, signal voids, and truncation artifacts. This also has a limiting impact on MR-based attenuation correction. Body parts and patient tissues exceeding the constraints of the limited transaxial FOV, e.g., the patient arms, may be displayed truncated, geometrically distorted, or as signal voids in the MR-based μ-maps. Such truncation artifacts in the μ-map may lead to a bias in PET quantification as PET signal-attenuating tissues are not correctly considered and displayed in MR-based AC.

The HUGE technique proposed by Blumhagen et al. [10*,*^19^] is an MR-based technique for truncation correction of peripheral body regions and patient arms. In the present study, the most recent implementation of the HUGE technique was used, employing a moving table acquisition that provides up to 60 cm left-to-right seamless MRI coverage for the left and right arms of patients [11]. The truncation-corrected areas of the right and left sides of the body are then merged with the four-compartment reference μ-map, hence providing AC of the central and peripheral body regions as further compartment (Fig. 1).

### Image analysis

Three different whole-body μ-maps were reconstructed for each patient: (1) the standard μ-map Dixon-based AC with 4-compartments serving as reference, (2) the 5-compartment μ-map, and (3) the 6-compartment μ-map (Fig. 1). All μ-maps and HUGE and MLAA raw data were inspected qualitatively for plausibility and artifacts. For each μ-map, it was visually examined how truncation correction with MLAA and HUGE performed in deriving the outer patient contours and to fill truncations along the arms with attenuation values.

To create three AC PET datasets for each patient, the raw PET data and the three different μ-maps were retrospectively reconstructed using the image data reconstruction tool provided by the PET/MR system manufacturer (e7 tools, Siemens Molecular Imaging, Knoxville, USA). The resulting three AC PET image datasets for all 23 patients and for each of the 28 examinations were assessed by an experienced radiologist (with 3 years of reading PET/MR) and by an experienced nuclear medicine specialist (with 6 years of reading PET/MR), and a consensus decision was made concerning the results. Image reading entailed identifying the primary tumor and/or lesion with up to five further lesions with focally increased radiotracer uptake in each patient, delineating the volume of interest (VOI) as well as computing the SUV_max_ in the PET datasets of each patient. Selecting criteria for up to 5 lesions per patient were highest SUV in the most visible bone lesions localized in major bones, such as pelvic bones, spine, thigh, and scapula. The VOIs around lesions were delineated using an automatic isocontour function with a fixed threshold of 40% of the SUV_max_ in the lesion. In order to ensure accurate and identical placement of all VOIs in all PET data reconstructions, VOIs were delineated with the help of a *syngo*.via workstation (Siemens Healthcare GmbH).

Relative difference images were calculated using MATLAB 2013b (MATrix LABoratory, MathWorks, Inc., Natick, MA, USA) to evaluate the quantitative difference between PET data corrected by four compartment and six-compartment μ-maps, as per the equation below.

$$ \mathrm{Relative}\ \mathrm{difference}\ \mathrm{maps}=\frac{\ \mathrm{PET}\ 6\ \mathrm{compartments}-\mathrm{PET}\ 4\ \mathrm{compartments}}{\mathrm{PET}\ 4\ \mathrm{compartments}}100\% $$

Bland−Altman plots were used to analyze the relative differences in the measured SUV_max_ values in all detected lesions. Descriptive statistics were used to calculate the mean values and standard deviation.

## Results

**Fig. 2:**
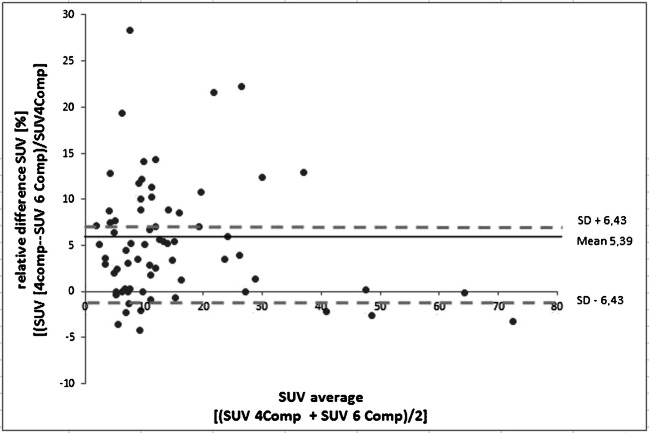
Bland–Altman plot shows the relative quantitative impact of the 6-compartment AC on SUV_max_ in 69 detected bone lesions when compared with the 4-compartment AC serving as reference. The mean increase in SUV_max_ of all 69 lesions is 5.4 ± 6.4%. The total range is − 4.3% up to 28.4% in individual lesions close to bone or truncations

Overall, 69 bone lesions were detected in all PET data reconstructions using three different AC methods for each patient examination. The Bland−Altman plot in Fig. 2 shows the relative differences between the standard 4-compartment and improved 6-compartment AC in the measured SUV_max_ of the 69 detected lesions. Note the overall gain in SUV_max_ due to the 6-compartment AC including bone and HUGE information. Considering all 69 lesions, the mean increase in SUV_max_ is 5.4 ± 6.4%. The total range was − 4.3% up to 28.4%. Maximal relative differences occurred in lesions close to bone or truncations.

Table 2 lists examples of six lesions that showed the highest quantitative impact on SUV_max_ due to application of improved AC. The highest relative increase in SUV_max_ with 28.4% could be observed in the thoracic spine (lesion no. 4). The second highest relative increase in SUV_max_ with 22.3% was observed in the os sacrum (lesion no. 3) of one patient. In these two examples, the relative impact of HUGE truncation correction on lesion SUV_max_ was higher than the relative impact of adding bone information (diff. 4-comp. vs. 5-comp.) to the μ-map. For the remaining four specific lesion examples, the relative quantitative impact of adding bone was higher than the relative impact of HUGE truncation correction.

**Table 2:** Six examples that each showed in a single lesion highest impact in relative difference of SUV_max_ between the standard 4-compartment and improved 5-compartment, respectively 6-compartment μ-map. Note that the highest relative increase in SUV_max_ with 28.4% could be observed in the thoracic spine (lesion no. 4). The second highest relative increase in SUV_max_ with 22.3% was observed in the os sacrum (lesion no. 3) of another patient. In these two examples, the relative impact of HUGE truncation correction on lesion SUV_max_ was higher than the relative impact of adding bone information (diff. 4-comp. vs. 5-comp.) to the μ-map

Lesion no.	Patient no.	Exam no.	Tracer	Lesion	Difference of SUV_max_:	Difference of SUV_max_:
					4-comp. vs. 5-comp (%)	4-comp. vs. 6-comp (%)
1	4	5	18F-FDG	Os acetabulum left	9.8	14.1
2	4	5	18F-FDG	Os sacrum	7.6	11.3
3	15	19	[68Ga]-PSMA	Os sacrum	6.3	22.3
4	16	20	[68Ga]-PSMA	Thoracic spine	3	28.4
5	23	28	124I-MIBG	Os acetabulum left	6.5	10.8
6	23	28	124I-MIBG	Os Ileum right	8.5	12.4

**Fig. 3:**
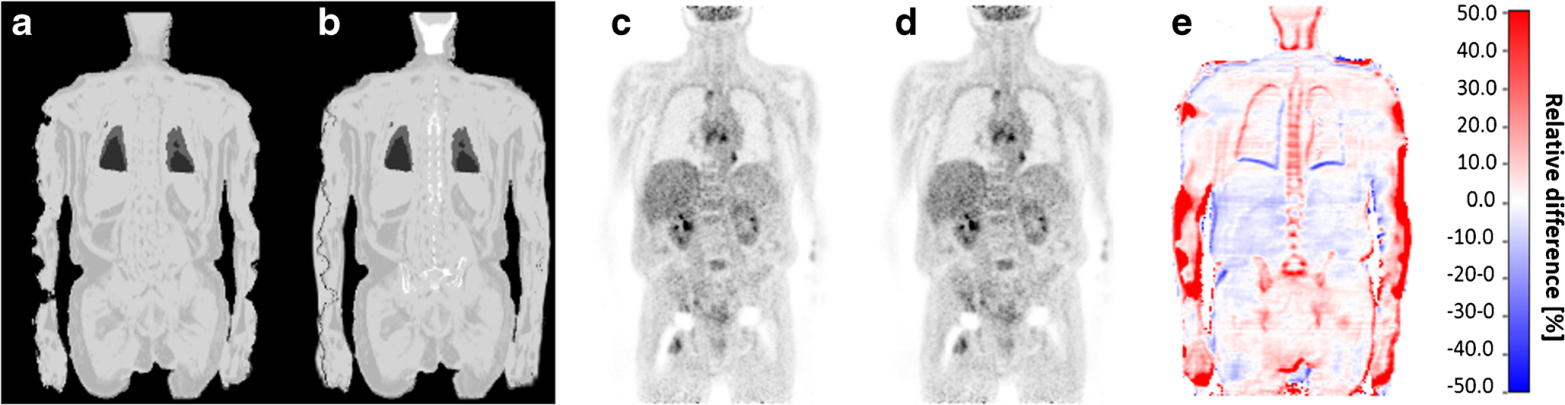
Patient example with injected ^18^F-FDG: standard 4-compartment μ-map serving as reference (**a**) and improved 6-compartment μ-map (**b**). **c** and **d** show corresponding attenuation-corrected PET data ((**c**) 4 compartments, (**d**) 6 compartments). **c** and **d** show no visible difference. **e** shows a calculated relative PET difference map, highlighting differences with color. The colored bar in (**e**) provides the quantitative bias of relative undercorrection (red) or overcorrection (blue) when comparing PET data corrected with 6-compartment μ-map to PET data corrected with a 4-compartment μ-map (reference). Maximal relative differences are observable in the previously truncated areas along the arms and in areas where bone was added to the μ-map

**Fig. 4:**
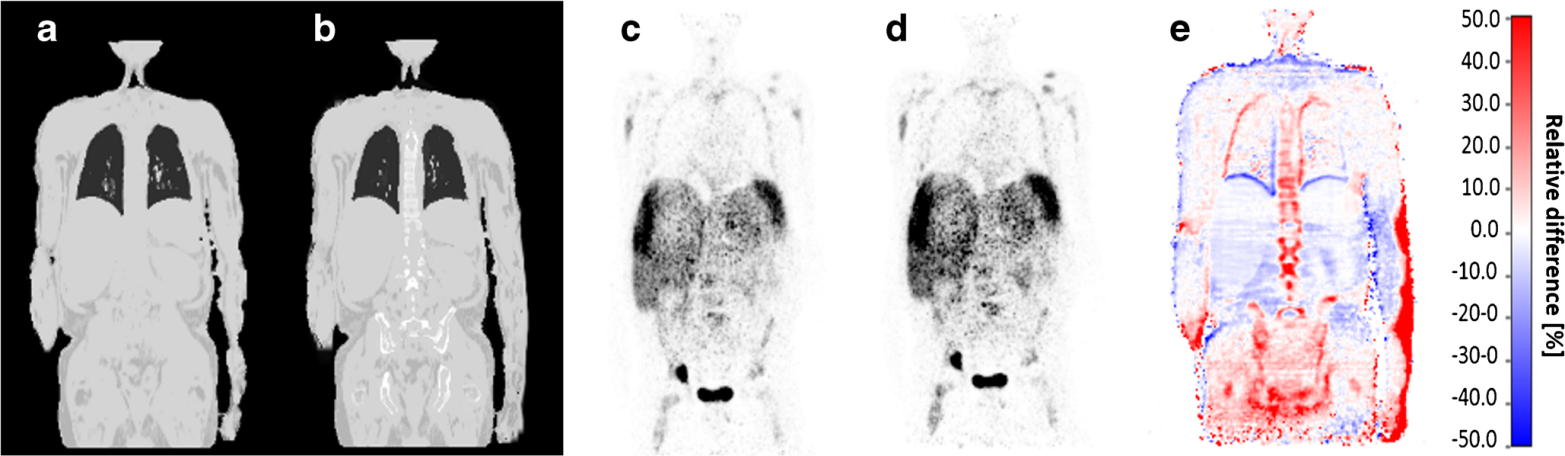
Patient example with injected ^68^Ga-DOTATOC: standard 4-compartment μ-map serving as reference (**a**) and improved 6-compartment μ-map (**b**) with corresponding attenuation corrected PET data (4-compartment (**c**), 6-compartment (**d**)). **e** A calculated relative PET difference map. Maximal relative differences between the 4- and 6-compartment AC PET data are observable in the truncated and bony areas also for this non-FDG tracer example

**Fig. 5:**
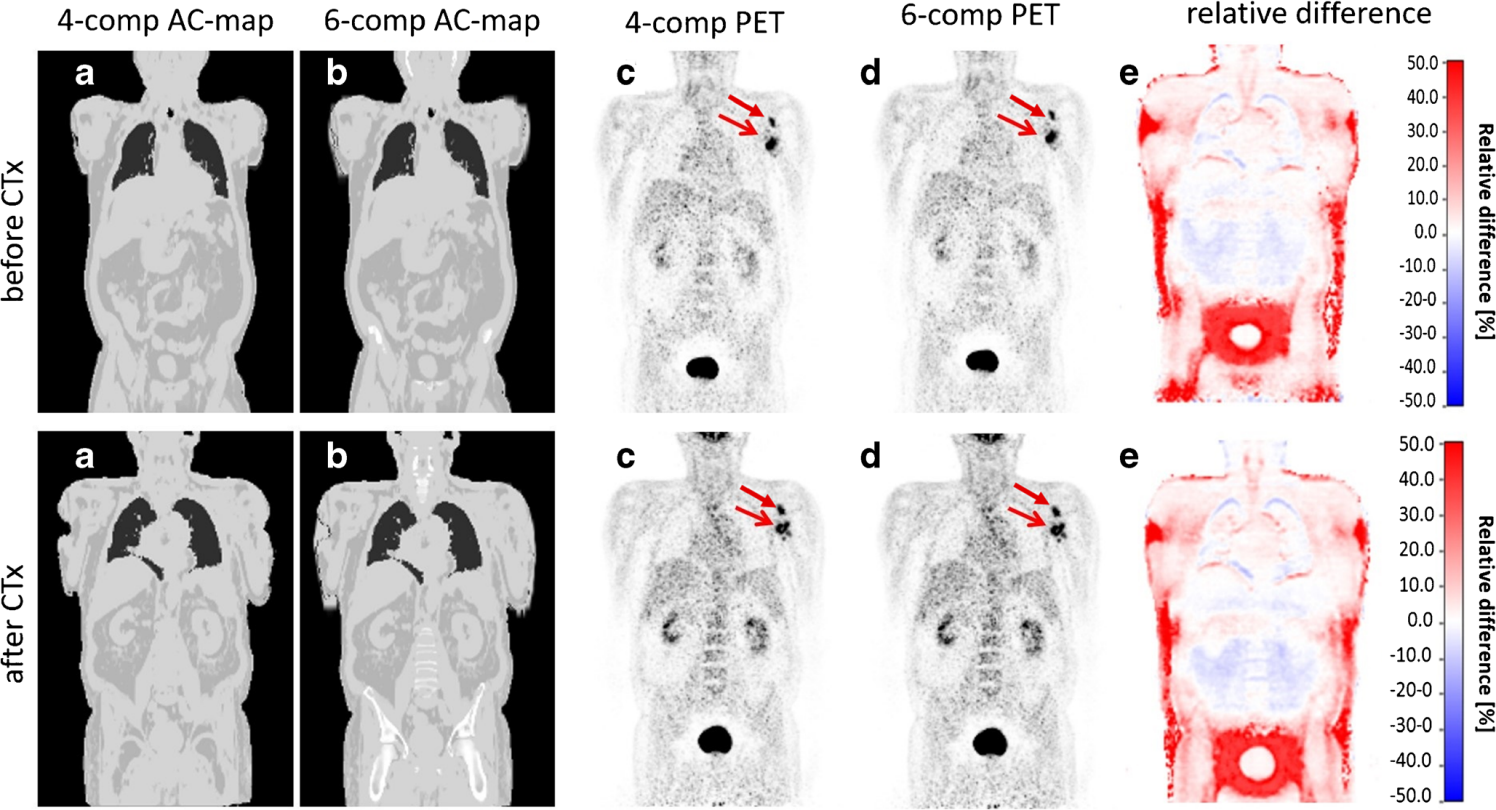
Patient case with injected ^18^F-FDG before and after chemotherapy (CTx): standard 4-compartment μ-map serving as reference (**a**) and improved 6-compartment μ-map (**b**) with corresponding corrected PET data to 4-compartments (**c**) and 6-compartments (**d**) with two marked lesions (red arrows) as well as calculated relative difference maps (**e**). The difference maps (**e**) again depict the quantitative differences between PET data reconstructed with the 4- and 6-compartment μ-maps. Note that all image panels (μ-maps, PET data, difference maps) show a high degree of comparability between both examinations before and after CTx, separated by 21 days, indicating high reproducibility. Quantitative results are provided in Table 3

Figures 3, 4, and 5 show the 4-compartment μ-map and the 6-compartment μ-map with corresponding corrected PET data and calculated relative PET difference images. In all patient examples, the improved μ-map works robust, independent of the choice of radiotracer (Figs. 3 and 4). HUGE truncation correction results in a realistic body contouring adding tissue volumes along the arm regions and major bones to the standard Dixon-VIBE AC map. The color bar of the difference maps (Figs. 3, 4, and 5) provides the quantitative bias in percentage of relative undercorrection (red) or overcorrection (blue) when compared to the reference 4-compartment μ-map. As to be expected, the largest differences (relative undercorrection by the 4-compartment μ-map, red) are located in regions that profit from the addition of the bone model and the addition of previously truncated areas along the patient’s arms. Blue color indicates relative overcorrection by the 4-compartment μ-map (blue) in regions that profit from improved scatter correction due to introduction of the 6-compartments. Namely, the thoracic and pelvic body regions show light blue areas indicating the effects of improved scatter correction.

One patient example is exhibited in Fig. 5 for a patient who had two PET/MR examinations before and after chemotherapy, separated by 21 days. Table 3 provides the measured SUV_max_ values of two bone lesions in this patient example for the 4-compartment μ-map and the 6-compartment μ-map with corresponding corrected PET data. Note that the relative impact of improved AC is highly comparable for each of the two lesions before and after chemotherapy. SUV_max_ of lesion no. 1 before chemotherapy increases by 5.4% due to 6-compartment AC while it increases by 5.2% after chemotherapy when comparing 6-compartment AC with 4-compartment AC as reference. Similar comparability can be seen for lesion no. 2, where the SUV_max_ increases by 8.9% before, and 10.0% following chemotherapy. In this patient example, the SUV_max_ of lesion no. 1 due to the impact of therapy decreased from 13.6 to 8.0 by 41.2%. The SUV_max_ of lesion no. 2 decreased from 14.7 to 9.9 by 32.6% between the two examinations.

**Table 3:** SUV_max_ values of two bone lesions measured in two exams (separated by 21 days), before and after chemotherapy (CTx) in a 18F-FDG patient example. PET data were corrected with a 4-compartment μ-map serving as the reference and with an improved 6-compartment μ-map. Note that the relative impact of the improved 6-compartment vs. the 4-compartment AC is highly comparable for each of the two lesions before and after chemotherapy, indicating a high repeatability for SUV_max_ quantification for each of the two lesions in two different examinations, separated by 21 days. Note also the quantitative impact of chemotherapy on both lesions, indicated by decreasing SUV_max_ values

Tracer FDG	SUV_max_ of bone lesion no. 1 (left glenoid)	SUV_max_ of bone lesion no. 2 (left glenoid)
4-Comp. before CTx	12.9	13.5
6-Comp- before CTx	13.6	14.7
4-Comp. vs. 6-comp. before CTx	5.4%	8.9%
4-Comp. 21 days after CTx	7.6	9.0
6-Comp. 21 days after CTx	8.0	9.9
4-Comp. vs. 6-comp. 21 days after CTx	5.2%	10.0%
6-Comp before CTx vs. 21 days after CTx	− 41.2%	− 32.6%

**Fig. 6:**
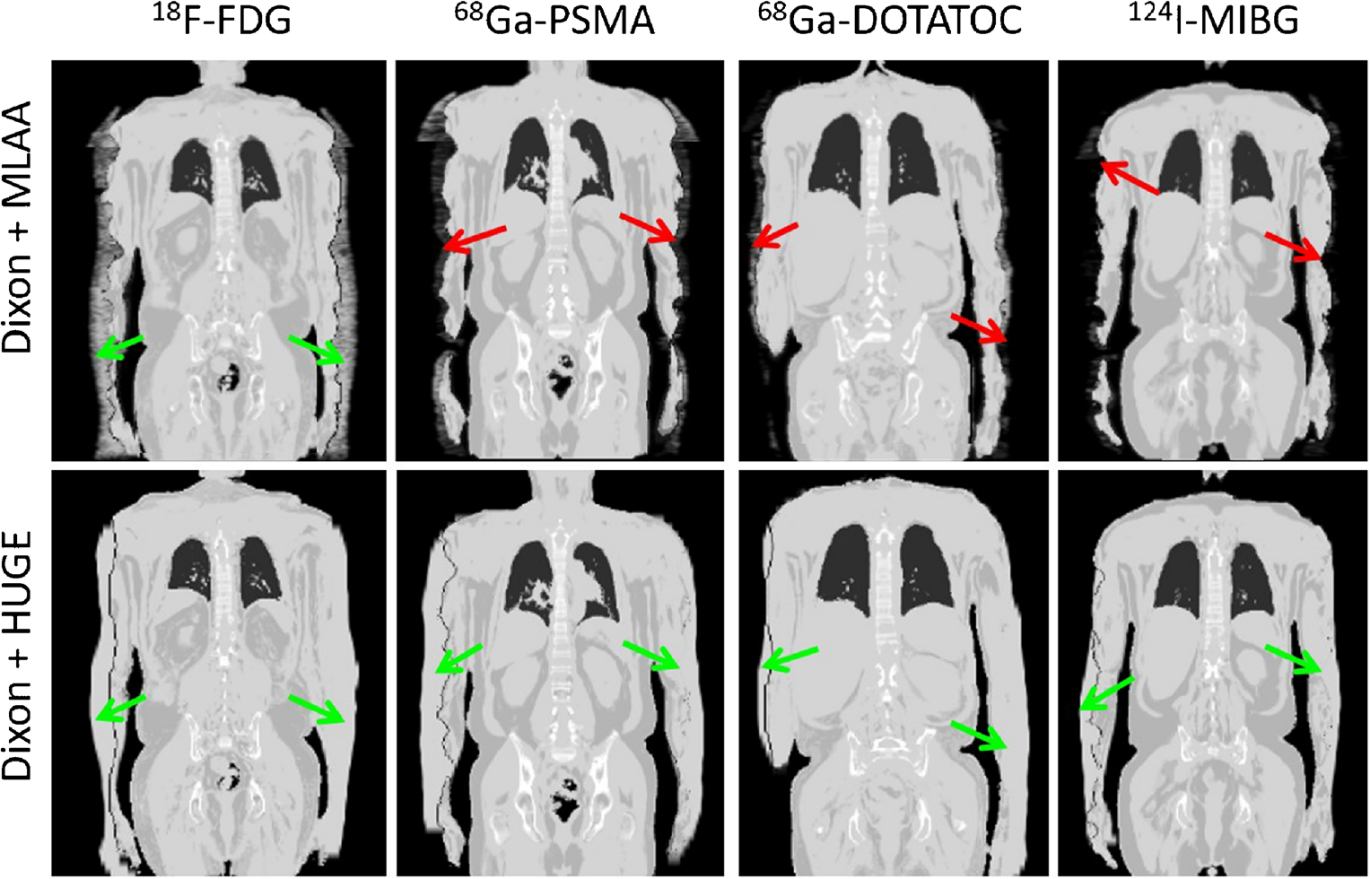
μ-maps with applied truncation correction due to PET-based MLAA (upper row) or MR-based HUGE (lower row) in four patient examples using four different radiotracers (^18^F-FDG, ^68^Ga-PSMA, ^68^Ga-DOTATOC, ^124^I-MIBG). Green arrows indicate accurate truncation correction, whereas red arrows indicate incomplete or failed truncation correction. Note that the MR-based HUGE method works robust, independent of the choice of radiotracer. MLAA provided acceptable truncation correction only in the patient example with ^18^F-FDG

Figure 6 shows that the MLAA method in PET/MR with radiotracers ^68^Ga-PSMA, ^68^Ga-DOTATOC, and ^124^I-MIBG failed to provide accurate contours for attenuation correction. Particularly in the ^124^I patient example, the MLAA method did not add any linear attenuation coefficient to the standard Dixon-VIBE AC map. MLAA in PET/MR with ^18^F-FDG works robust, but reveals slight overestimations along the patients’ arms (green arrow). The truncation correction based on HUGE reveals a robust application along the patients’ arms with all 4 radiotracers and in all 23 patients and 28 examinations.

## Discussion

This retrospective study of 28 PET/MR examinations aimed to evaluate the quantitative effect of an improved MR-based AC method, including bone segmentation and HUGE truncation correction, in PET/MR whole-body hybrid imaging, specifically on 23 oncology patients with bone metastasis, using ^18^F-FDG, ^68^Ga-PSMA, ^68^Ga-DOTATOC, and ^124^I-MIBG radiotracers. In total, 69 bone lesions were detected and included in the evaluation. The overall gain in SUV_max_ due to application of improved AC for all 69 lesions was 5.4 ± 6.4%, the total range was − 4.3% up to 28.4%. These quantitative results are in good accordance with the results of previously published studies on the impact of AC methods in whole-body PET/MR [11*,*^12^].

The average increase of SUV_max_ by 5.4 ± 6.4% and the total range of − 4.3% up to 28.4% when applying the 6-compartment μ-maps for AC indicates that PET data reconstructed with the standard 4-compartment μ-maps underestimates the tracer uptake in lesions, and, more specifically in bone lesions, which was one of the major subjects of the present study. These results are in good accordance with the findings reported by Samarin et al. [5]. In their study, conducted on PET/CT patient data, Samarin et al. simulated and examined the quantitative impact of substituting μ values of bone tissue in μ-maps by the lower μ values of soft tissue, as it is also the case with the standard 4-compartment μ-maps in PET/MR hybrid imaging [5]. As to be expected, the highest quantitative impact in their study was found for bone lesions, while other organ and soft tissue lesions did show only lower quantitative impact [5].

Likewise, the addition of truncation correction to the 4-compartment standard μ-maps had measureable quantitative impact on the increase of SUV_max_ of selected lesions in this study. This has also been demonstrated in previous studies by Blumhagen et al. [10], by Lindemann et al. [11], and by Oehmigen et al. [12]. In the present study, the highest impact of HUGE truncation correction on PET was quantified in lesions close to the arms. However, in the remainder of the body (e.g., thorax, shoulders, and hips), also bias in PET activity quantification was measurable. Accordingly, the relative difference images (Figs. 3 and 4) show that the relative impact of improved AC is mostly increased in the thoracic and pelvic region. Adding volumes of soft tissue in patients’ upper arms relative to the less attenuating large volume of lung tissue results in substantial relative gains of total tissue attenuation in the thoracic region. Also the pelvic region benefits from extended MR FOV due to HUGE because of the patients’ arm posture in PET/MR. Here, HUGE adds tissue volume along the patients arms to the μ-map in previously truncated regions. In addition, the 5-compartment μ-map adds the pelvic bones and the femurs in the pelvis, as well as the spine in the thorax to the μ-map. Thus, major bones with relatively high attenuation values are added to these regions. Consequently, the six lesions that showed highest relative difference of SUV_max_ due to the impact of adding bone tissue and truncation correction to the MR-based μ-maps were located in both of these body regions: the thorax and the pelvis (Table 2).

Calculated difference maps between 4 compartment and 6 compartment on Figs. 3, 4, 5, and 6 exhibit a relative undercorrection of PET signal in the 4-compartment μ-maps due to missing bone information and truncated arms (red color). The blue areas in the difference maps (e.g., in the thorax and pelvis) are the results of improved scatter correction when applying the 6-compartment AC as also reported in the studies by Lindemann et al. [11] and by Oehmigen [12]. Scatter correction in PET/MR is also based on the AC maps. Thus, the incorporation of the bone model and information from extended MR FOV in the 6-compartment AC map also affects scatter correction from these two additional compartments. Therefore, this results in a more realistic scatter estimation when applying the improved 6-compartment μ-maps (blue color).

In recent clinical studies on whole-body PET/MR investigating oncologic patients injected with ^18^F-FDG as radiotracer, it was shown that improved AC including a bone model and methods for truncation correction showed robust results and MR-based truncation correction provided realistic body contours [11*,*^12^*,*^20^]. In contrast to these previous studies, the improved AC method in the present study was applied to a patient collective with bone lesions and that was injected with four different radiotracers (^18^F-FDG, ^68^Ga-DOTATOC, ^68^Ga-PSMA, and ^124^I-MIBG). One major finding of this present study is that the MR-based HUGE truncation correction method provided realistic and robust body contours for each patient included in this study, independent of the choice of radiotracer (Fig. 6). This is important to note, since the previous standard method for truncation correction, namely PET-based MLAA correction, failed in deriving body contours from PET images for all patients in this study that were investigated with non-FDG radiotracers. This is because the PET-based MLAA algorithm in the non-FDG examinations did not detect sufficient signal from unspecific tracer accumulation in tissue to derive body contours. Reported data by Lindemann [11] demonstrate the same limitations of MLAA in patients using ^124^I-iodine.

The PET/MR system used in this study has no time-of-flight (TOF) PET detector capability. Therefore, the implementation of the MLAA algorithm evaluated in this study may not provide ideal results since it is assumed that the MLAA technique benefits from TOF capability [21]. In previous studies, it has been shown that PET/MR systems with TOF capability in select cases improve the overall capability to reduce artifacts in the μ-map caused by implants [22], and may improve lesion detection and quantification in PET/MR studies when compared to non-TOF detection [15]. Furthermore, improvements in truncation correction [23] and in MLAA reconstruction [15] have been demonstrated when TOF PET detection was additionally applied. These studies suggest that TOF potentially provides improved performance of the MLAA algorithm when applied for truncation correction, although this has not been explicitly demonstrated according to the literature [15*,*^22^–^25^]. On the other hand, the MR-based HUGE method on the available Biograph mMR system in this study provided robust truncation correction in all patients, independent of the radiotracer applied. Truncation correction with HUGE could be successfully applied in each patient independent of the choice of radiotracer. In each Dixon-VIBE-based μ-map, arm volume could be accurately added, and no arm was missing due to a failed contour segmentation or bias in the HUGE MR raw data. Thus, we conclude that the HUGE method worked reliably, independent of the choice of radiotracer.

There is high interest in ^68^Ga-PSMA PET for staging, therapy, and follow-up of prostate cancer; however, the high specificity of the radiotracer in prostate imaging frequently presents challenges [26*,*^27^]. The detection of bone lesions when using the radiotracer ^68^Ga-PSMA in prostate cancer imaging may be limited [26*,*^28^]. In a study by Freitag et al. [29], it has been found that bone lesions tend to show lower levels of PSMA expression than, e.g., lesions in lymph nodes [29]. While this finding may also depend on biological behavior of tracer metabolism and lesion size, an accurate μ-map also considering the attenuation due to bone tissue may help to improve diagnostic assessment even in faint bone lesions. A second associated challenge is that ^68^Ga-PSMA during the course of a PET/MR examination concentrates in the urinary bladder and may lead to photopenic artifacts, also called halo artifacts [30–32], surrounding the bladder. Such halo artifacts may obscure lesions or may lead to a bias in PET quantification of pelvic lesions [30–32]. Inaccurate scatter correction (SC) has been identified as a significant factor in the origin and extent of halo artifacts. SC algorithms in PET/MRI also rely on an accurate μ-map of the patient tissues. In particular, the frequently occurring truncations along the arms in the μ-map due to a limited MRI FOV seem to be an important factor in the occurrence of the halo artifact, as has been shown by Afshar et al. [31]. This observation is supported by the study of Noto et al. [32] who showed that filling the truncated and missing parts along the arms in the Dixon-VIBE μ-map using the MLAA method resulted in reduced halo artifact size in 68Ga-PSMA PET/MR hybrid imaging [32]. In this context, applying the recent HUGE method for truncation correction may also reduce halo artifacts, as this has been demonstrated in a study investigating the impact of scatter correction and HUGE on halo artifact size in 68Ga-PSMA PET/MR of the prostate [33].

Accuracy and repeatability of MR-based attenuation correction are important methodological preconditions to quantify the effect of therapy on specific tumors and lesions in a clinical setting. In this context, the results of Table 3 demonstrate accurate quantification of the effect of therapy on lesion activity. The results indicate a high repeatability of SUV_max_ quantification for each of the two lesions in two different examinations, separated by 21 days. Although applying the improved AC methods in this specific patient case did not have a direct clinical impact on patient or therapeutic management, applying the most accurate AC methods available is an important methodological precondition, as whole-body PET/MR hybrid imaging today is increasingly used as diagnostic hybrid imaging modality for monitoring of therapeutic responses [34*,*^35^].

In total, 17 PET/MR examinations with ^18^F-FDG and 11 with non-^18^F-FDG tracers were conducted. From 69 detected bone lesions, 39 lesions were measured in patients injected with ^18^F-FDG, and 30 bone lesions were found in the patients with non-^18^F-FDG tracers. The comparable low number of non-FDG tracers may be seen as a limitation of this study; however, the impact of μ-map performance on patient data acquired with different tracers could be demonstrated in all of these examples. Finally, for all lesions a relative quantitative impact of μ-map performance was measureable; however, the relative changes in SUV_max_ in the present study were only minor and, thus, had no clinical impact on lesion detectability and on therapeutic management.

## Conclusion

The addition of bone segmentation and truncation correction as fifth and sixth compartment to a standard 4-compartment MR-based μ-map improves attenuation correction in whole-body PET/MR on patients with bone lesions and using various radiotracers. The MR-based HUGE method for truncation correction worked robust, independent of the radiotracer used. Improved MR-based AC is important to ensure best possible diagnostic image quality and accurate PET quantification in whole-body PET/MR studies with various radiotracers and when PET/MR is used for monitoring of therapeutic response.
